# Artificial Intelligence for Radiation Dose Optimization in Pediatric Radiology: A Systematic Review

**DOI:** 10.3390/children9071044

**Published:** 2022-07-14

**Authors:** Curtise K. C. Ng

**Affiliations:** 1 Curtin Medical School, Curtin University, GPO Box U1987, Perth, WA 6845, Australia; curtise.ng@curtin.edu.au or curtise_ng@yahoo.com.hk; Tel.: +61-8-9266-7314; Fax: +61-8-9266-2377; 2 Curtin Health Innovation Research Institute (CHIRI), Faculty of Health Sciences, Curtin University, GPO Box U1987, Perth, WA 6845, Australia

**Keywords:** as low as reasonably achievable, computed tomography, convolutional neural network, deep learning, dose reduction, generative adversarial network, image processing, machine learning, medical imaging, noise

## Abstract

Radiation dose optimization is particularly important in pediatric radiology, as children are more susceptible to potential harmful effects of ionizing radiation. However, only one narrative review about artificial intelligence (AI) for dose optimization in pediatric computed tomography (CT) has been published yet. The purpose of this systematic review is to answer the question “What are the AI techniques and architectures introduced in pediatric radiology for dose optimization, their specific application areas, and performances?” Literature search with use of electronic databases was conducted on 3 June 2022. Sixteen articles that met selection criteria were included. The included studies showed deep convolutional neural network (CNN) was the most common AI technique and architecture used for dose optimization in pediatric radiology. All but three included studies evaluated AI performance in dose optimization of abdomen, chest, head, neck, and pelvis CT; CT angiography; and dual-energy CT through deep learning image reconstruction. Most studies demonstrated that AI could reduce radiation dose by 36–70% without losing diagnostic information. Despite the dominance of commercially available AI models based on deep CNN with promising outcomes, homegrown models could provide comparable performances. Future exploration of AI value for dose optimization in pediatric radiology is necessary due to small sample sizes and narrow scopes (only three modalities, CT, positron emission tomography/magnetic resonance imaging and mobile radiography, and not all examination types covered) of existing studies.

## 1. Introduction

Radiology is an indispensable part of modern healthcare. However, most of the medical imaging modalities, such as computed tomography (CT), positron emission tomography (PET), and general radiography, use ionizing radiation for image production [1,2,3,4,5,6,7,8,9,10,11,12,13,14,15,16]. Although the radiation dose involved in these imaging modalities is low (<100 mSv), and their real risk is unclear, some epidemiologic and biologic studies have demonstrated that these radiological examinations can cause cancers [17,18,19,20,21,22,23]. Hence, “as low as reasonably achievable” (ALARA) has become the fundamental principle of radiology practice [17,24,25]. International Commission on Radiological Protection (ICRP) has introduced the diagnostic reference levels (DRLs) initiative for radiological departments to identify examinations with radiation doses exceeding their corresponding DRLs and trigger the radiation dose-optimization process [26,27,28,29,30,31,32]. As the radiation used in radiological examinations is the source of signal, a reduction of the radiation amount results in a decrease of signal strength and an increase of image noise. Traditionally, the dose-optimization process involves the manipulation of a range of exposure/scan parameters and identification of parameters that deliver the lowest radiation dose but still producing images able to meet minimal diagnostic requirements [33,34,35,36]. Since the introduction of digital medical imaging, image processing has played an important role in the radiation dose optimization [37,38,39]. However, typical image processing techniques are unable to overcome the tradeoff between image noise and spatial resolution [9,10,11,12]. For the last few years, artificial intelligence (AI) has been introduced into radiology for radiation dose optimization. Studies have demonstrated its ability in pushing the limit, i.e., able to further reduce the radiation dose but without sacrificing image quality, such as noise and spatial resolution [1,6,9,10,11,12,15,16].

The dose optimization is particularly important for pediatric patients because they have longer life and more rapid cell proliferation, leading to two to three times more susceptibility to the potential harmful effects of ionizing radiation than the adult counterpart [17,33,36,40]. Nonetheless, dose optimization in pediatric radiology is challenging, as there is a greater variation of body size and composition within and across age groups [4,33]. Despite being an important and challenging topic area, apparently, only one narrative review article on this area has been published yet, and it is about deep learning (a subset of AI) image reconstruction (DLIR) for dose optimization in pediatric CT [17]. Hence, it is timely to conduct a systematic review about the use of AI for dose optimization in pediatric radiology. The purpose of this article is to systematically review published original studies to answer the question “What are the AI techniques and architectures introduced in pediatric radiology for dose optimization, their specific application areas, and performances?”

## 2. Materials and Methods

This systematic review on the AI for radiation dose optimization in pediatric radiology was conducted as per the PRISMA guidelines and patient/problem, intervention, comparison, and outcome (PICO) model [41,42]. This involved literature search, article selection, and data extraction and synthesis.

### 2.1. Literature Search

The literature search with use of electronic scholarly publication databases, including *Google Scholar, PubMed/Medline, ScienceDirect, Scopus*, and *Web of Science* was conducted on 3 June 2022 to identify articles about the AI for dose optimization in pediatric radiology published between 2017 and 2022. The search statement used was (“Artificial Intelligence” OR “Machine Learning” OR “Deep Learning”) AND (“Dose Optimization” OR “Dose Reduction”) AND (“Pediatric” OR “Children”) AND (“Radiology” OR “Medical Imaging”). The keywords used in the search were based on the review focus. The year range was determined based on a narrative review about current and future applications of AI in radiology, which showed the use of AI for dose optimization in radiology not evident before 2017 [43].

### 2.2. Article Selection

A reviewer with more than 20 years of experience in conducting literature review was involved in the article selection process [44]. Only peer-reviewed original research articles that were written in English and focused on the use of AI for dose optimization in pediatric radiology were included. Grey literature, conference abstracts, editorials, review, perspective, opinion, commentary, and non-peer-reviewed (e.g., those published via the arXiv research-sharing platform, etc.) articles were excluded because of the following reasons: Well-established methodological guidelines were not available for proper selection of grey literature. Conference abstracts could not provide complete study information. Only secondary information was presented in editorials, review, perspective, opinion, and commentary articles. Non-peer-reviewed articles might provide unsubstantiated information [45,46].

**Figure 1:**
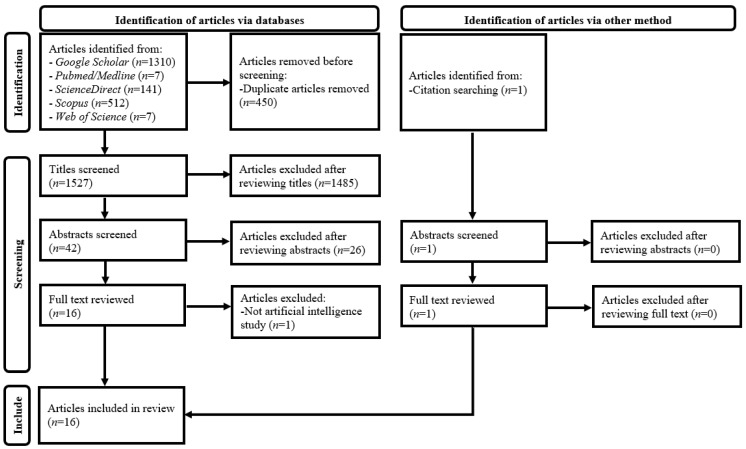
PRISMA flow diagram for systematic review of artificial intelligence for radiation dose optimization in pediatric radiology.

Figure 1 illustrates details of the article selection process [41]. A three-stage screening process through assessing (1) article titles, (2) abstracts, and (3) full texts against the selection criteria was employed after duplicate article removal from results of the database search. Every non-duplicate article within the search results was retained until its exclusion could be decided. Lists of references of the included papers were reviewed for additional, relevant article identification [46].

### 2.3. Data Extraction and Synthesis

A data extraction form (Table 1) was developed based on a recent systematic review on the use of AI in radiology [45]. The data, including names and countries of authors, publication years, clinical domains (radiology/nuclear medicine), AI techniques (such as machine learning and deep learning (DL)), model architectures (e.g., convolutional neural network (CNN), generative adversarial network (GAN), etc.), specific application areas (i.e., examination types and approaches that AI was used to achieve dose optimization), imaging modalities, details of AI model development (i.e., whether homegrown or commercially available model and arrangement of model training and testing), AI model evaluation approach (e.g., phantom study, clinical study, etc.), and key findings of AI model performance in dose optimization (including figures of dose reduction and diagnostic values and subjective and objective image assessment scores), were extracted from each included paper. To facilitate comparison of the AI model performance, percentage of dose reduction (if not reported) was synthesized based on the available absolute dose figures. When multiple image-quality-related figures were reported in a study, the most clinically relevant figures were presented. Diagnostic values were considered the most clinically relevant performance figures, while the objective image assessment scores were determined least relevant [47,48]. Quality assessment scores were determined for all included articles based on the quality assessment tool for studies with diverse designs (QATSDD) and expressed as percentages [49]. Less than 50%, 50–70%, and greater than 70% were considered low, moderate, and high study quality, respectively [46].

## 3. Results

Sixteen articles met the selection criteria and were included in this review. Table 1 shows these study characteristics [1,2,3,4,5,6,7,8,9,10,11,12,13,14,15,16]. All but one article were published in the last two years, representing that the AI for dose optimization in pediatric radiology has only just become popular [1,2,4,5,6,7,8,9,10,11,12,13,14,15,16]. Nearly all (14) studies were determined high quality [1,4,5,6,7,8,9,10,11,12,13,14,15,16], and the lower quality ones were “pure” phantom studies [2,3]. All studies used the DL technique [1,2,3,4,5,6,7,8,9,10,11,12,13,14,15,16] and were conducted by 12 groups of researchers from USA (*n* = 8) [1,8,9,10,11,12,13,14], People’s Republic of China (*n* = 6) [8,9,10,11,12,16], Republic of Korea (*n* = 5) [2,3,5,7,15], Germany (*n* = 2) [4,14], and Japan (*n* = 1) [6]. Only two studies were about nuclear medicine (whole-body PET/magnetic resonance imaging (MRI)) [13,14]. For the 14 radiology-related studies [1,2,3,4,5,6,7,8,9,10,11,12,15,16], all except one were related to CT, covering body parts such as the abdomen (*n* = 10) [1,2,3,4,5,6,7,8,15,16], chest (*n* = 8) [1,4,8,9,10,11,15,16], head (*n* = 1) [12], neck (*n* = 1) [8], and pelvis (*n* = 1) [1], and four focused on CT angiography [8,9,10,11] as well as one on dual-energy CT (DECT) [5]. Thirteen studies (81.3%) used commercially available AI models for dose optimization (TrueFidelity, General Electric Healthcare (GE): *n* = 7 [8,9,10,11,12,15,16]; AiCE, Canon Medical Systems: *n* = 3 [1,2,6]; ClariCT.AI, ClariPI: *n* = 1 [5]; SimGrid, Samsung Electronics Co., Ltd.: *n* = 1 [4]; SubtlePET 1.3, Subtle Medical: *n* = 1 [13]). Ten studies (62.5%) employed DLIR for CT dose optimization as a result of the dominance of GE TrueFidelity and Canon AiCE with the CNN architecture [1,2,6,8,9,10,11,12,15,16]. Hence, the CNN was the most popular (87.5%) AI architecture among the included studies [1,2,4,5,6,8,9,10,11,12,13,14,15,16].

Clinical evaluation of the AI model performance was conducted in all but two studies [1,4,5,6,7,8,9,10,11,12,13,14,15,16], and the use of phantom for an additional evaluation was also noted in three (21.4%) of the clinical studies [6,15,16]. Collectively, these clinical studies covered pediatric patients aged from 0 to 18 years [1,4,5,6,7,8,9,10,11,12,13,14,15,16]. All except one study recruited less than 100 patients for the model evaluation [1,5,6,7,8,9,10,11,12,13,14,15,16]. The only exception had 134 patients [4]. A retrospective approach was employed in about two-thirds (9 out of 14) of the clinical studies [1,4,5,6,7,8,9,12,15]. About 70% of (11) included studies reported absolute dose figures and/or dose reduction percentages. The performance of dose reduction of the AI models with acceptable image quality ranged from 11% to 95% [1,2,5,6,7,10,11,12,13,14,16]. More than half (6) of these studies reported that their AI models were able to achieve dose reductions between 36% and 70% [1,6,7,10,13,16] although three other studies showed dose reductions between 85% and 95% [2,12,14], and another two showed 11–20% dose reductions [5,11]. For the two most popular commercial AI models, GE TrueFidelity and Canon AiCE, great variations of their dose reduction performances, namely 11–85% and 52–95%, were noted, respectively [1,2,6,10,11,12,16].

## 4. Discussion

The findings of this systematic review on the AI for radiation dose optimization in pediatric radiology are consistent with several recent narrative reviews about the use of AI in radiology [17,43,51]. For the narrative review about the current and future applications of AI in radiology published in 2018 [43], only one study regarding low-dose CT denoising published in 2017 was cited [52]. However, recently, more studies about the use of AI for dose optimization have been published, resulting in a narrative review about the AI for dose optimization in pediatric CT available in 2021 [17]. This demonstrates that the use of AI for dose optimization in pediatric radiology has attracted the attention of the profession recently. That narrative review indicated the DLIR allowed 30–80% dose reduction in pediatric CT but was still able to produce images with diagnostic quality. This systematic review with inclusion of more studies about dose optimization in pediatric CT and covering other imaging modalities shows that the majority of the AI models were able to reduce the radiation dose by 36–70% [1,6,7,10,13,16]. Nonetheless, three studies included in this systematic review demonstrated that the use of AI could achieve further radiation dose reduction (up to 95%) [2,12,14]. Apparently, the large variation of dose reduction performances is due to the retrospective nature of many included studies [1,4,5,6,7,8,9,12,15], which did not allow further manipulation of examination/scan parameters to obtain ultra-low-dose images for evaluating whether the AI models could restore the quality of these ultra-low-dose images to close to the original [9]. Although there is a greater flexibility for phantom studies to manipulate the examination/scan parameters without any ethical and radiation dose concerns, enabling further exploration of the potential of these AI models, their evaluation outcomes tend to be less clinically relevant [47,48]. For example, Jeon et al. [2] reported that Canon AiCE was able to reduce the CT dose by 95% with the contrast-to-noise ratio values of the DLIR phantom images similar to those reconstructed by filtered back projection, but it is unclear whether these findings could be translated into clinical practice exactly. Nonetheless, Wang et al.’s [14] clinical prospective study showed that their homegrown AI denoising model developed through transfer learning with the use of 17 standard-dose PET simulated 6.25% ultra-low-dose PET, and MRI training datasets were able to reduce the radiation dose by 93.8% for the whole-body PET examinations with adequate diagnostic accuracy. This implies that it is feasible to use the AI denoising to achieve about 90% dose reduction in the clinical practice although all included studies had small sample sizes and/or number of training datasets [1,4,5,6,7,8,9,10,11,12,13,14,15,16], which is a common issue of AI studies in radiology due to limited availability of medical images [53]. Nevertheless, through the use of transfer learning (i.e., retraining an existing AI model using a smaller number of datasets with or without modification of its architecture) to develop an AI model for performing a similar task, such a model could provide a dose-optimization performance comparable to commercially available models (e.g., Canon AiCE, GE TrueFidelity, etc.) trained with more datasets [2,12,14,43].

It is within expectation that all but two studies used the AI models with the deep CNN architecture because the CNN architecture emerged in 1980s, and hence, it has been widely used in radiology, with satisfactory performances well-demonstrated [1,2,4,5,6,8,9,10,11,12,13,14,15,16,37]. However, one included study published in 2022 employed the more recent and advanced DL architecture: GAN, which was designed in 2014 [7,51]. According to a narrative review about the use of GAN in radiology published in 2021 [51], the CNN-based denoising models could cause CT images having a plastic-like appearance, which is similar to those produced by iterative reconstruction due to over-smoothing. In contrast, the GAN is a more complex architecture with a generator and a discriminator, which requires simultaneous training of these two, increasing the complexity of model development [37]. Nonetheless, the GAN-based denoising models are able to preserve texture details and hence produce images with quality matching standard images [51]. The GAN-based dose-optimization study included in this systematic review also demonstrated similar findings that their readers were unable to differentiate between the standard-dose and GAN-processed images although only 36.6% dose reduction was achieved in their study [7]. Another non-CNN-based dose-optimization study included in this review employed the Gaussian mixture model (GMM) architecture [3]. The use of GMM for medical image denoising was reported before the emergence of GAN [54]. However, it is not widely adopted in radiology, and its clinical performance in pediatric radiology dose optimization remains unclear [3,17,43,51].

This paper is the first systematic review on the AI for radiation dose optimization in pediatric radiology covering the imaging modalities of CT, PET/MRI, and mobile radiography and hence advancing the previous narrative review on the AI for dose optimization in pediatric CT published in 2021 [17]. Although it is well-known that radiation dose burden is a significant issue in pediatric CT [1,2,3,4,5,6,7,8,9,10,11,15,16], the dose involved in a PET scan is comparable to that of a CT examination [14]. Furthermore, general radiography is the most common radiological examination type for pediatric patients despite being a low-dose modality [36]. Nonetheless, as per the ALARA principle, the value of AI for dose optimization in other modalities that use ionizing radiation for pediatric examinations should be explored in the future [17,24,25]. Moreover, given the relatively narrow focus and small sample size of the included studies, future studies on this topic area for CT, PET, and general radiography need to have greater scale and wider scope [1,4,5,6,7,8,9,10,11,12,13,14,15,16]. Besides, further exploration of the use of GAN for dose optimization appears warranted [7,51].

This systematic review has two major limitations. Article selection, data extraction, and synthesis were performed by a single author, albeit one with more than 20 years of experience in conducting literature reviews. According to a recent methodological systematic review [44], this is an appropriate arrangement provided that the single reviewer is experienced. Additionally, through adherence to the PRISMA guidelines and the use of the data extraction form (Table 1) devised based on the recent systematic review on AI in radiology and QATSDD, the potential bias should be addressed to certain extent [41,45,46,49]. In addition, only articles written in English and published within last five years were included, potentially affecting comprehensiveness of this systematic review. Nevertheless, this review still has a wider coverage than the previous narrative review on the AI for dose optimization in pediatric CT [17].

## 5. Conclusions

This systematic review shows that the deep CNN was the most common AI technique and architecture used for radiation dose optimization in pediatric radiology. All but three included studies evaluated the AI performance in dose optimization of abdomen, chest, head, neck, and pelvis CT; CT angiography; and DECT through DLIR. The majority of studies demonstrated that the AI could reduce radiation dose by 36–70% without losing diagnostic information. Despite the dominance of commercially available AI models based on the deep CNN, the homegrown models, including the one with the more recent and advanced architecture, i.e., GAN, could provide comparable performances. Future exploration of the value of AI for dose optimization in pediatric radiology is necessary, as the sample sizes of the included studies appear small, and only three imaging modalities, namely CT, PET/MRI, and mobile radiography, rather than all examination types were covered.

## Figures and Tables

**Table 1:** Study characteristics of artificial intelligence for radiation dose optimization in pediatric radiology.

Author, Year, and Country	Clinical Domain	AI Technique and Architecture	Application Area for Dose Optimization	Imaging Modality	AI Model Development	AI Model Evaluation Approach	Key Findings of AI Model Performance
Brady et al. (2021), USA [1]	Radiology	DL-Convolutional neural network	DLIR of contrast-enhanced pediatric chest-abdomen-pelvis CT	CT	Commercially available model (AiCE, Canon Medical Systems, Tochigi, Japan) trained by image pairs of lower-dose CT with HIR and high-dose CT with MBIR and tested with datasets not involved in the training	Retrospective clinical study involving 19 children (mean age: 11 ± 5 y; range: 3–19 y)	With SBIR as reference, 52% dose reduction with noise texture and spatial resolution maintained, highest radiologists’ confidence rating (scale 1–10) among 4 approaches (DLIR: 7 ± 1; SBIR & MBIR: 6.2 ± 1; FBP: 4.6 ± 1), and object detectability improved by 51%, 18%, and 11% when compared with FBP, SBIR, and MBIR, respectively.
Jeon et al. (2022), Republic of Korea [2]	Radiology	DL-Convolutional neural network	DLIR of non-contrast pediatric abdominal CT	CT	Commercially available model (AiCE, Canon Medical Systems) trained by image pairs of lower-dose CT with HIR and high-dose CT with MBIR and tested with datasets not involved in the training	Phantom study involving phantoms with diameters, 16 (pediatric) and 32 cm (adult)	For 80–120 kV, CTDI_vol_ of DLIR images of pediatric phantom with CNR similar to corresponding FBP images was 5% of counterpart, representing 20-fold dose reduction potential.
Kim et al. (2017), Republic of Korea [3]	Radiology	DL-Gaussian mixture model	Post-processing of non-contrast pediatric abdominal CT images	CT	Homegrown model without training and testing details disclosed	Phantom study involving PMMA phantoms with diameters 12, 16, 20, 24, and 32 cm	Contrast-to-noise ratio dose increase by 1.7–4.9 times and 1.6–4.2 times for settings of 80–140 kV and fixed-tube current of 200 mA and 50–300 mA and fixed-tube potential of 120 kV, respectively.
Krueger et al. (2022), Germany [4]	Radiology	DL-Convolutional neural network	Post-processing of pediatric mobile chest and abdominal X-ray images acquired in intensive care units	Mobile radiography	Commercially available model (SimGrid, Samsung Electronics Co., Ltd., Suwon-si, Republic of Korea) trained by 30,000 images	Retrospective clinical study involving 210 images of 134 children (mean age: 4.2 y; range: 0–18 y)	Subjective image quality assessment demonstrated significant image quality improvement for patients with weight greater than 10 kg (odds ratio = 6.68, *p* < 0.0001), indicating its dose reduction potential.
Lee et al. (2021), Republic of Korea [5]	Radiology	DL-Convolutional neural network	Post-processing of pediatric abdominal DECT with lower CM concentration and noise-optimized virtual monoenergetic IR	CT	Commercially available model (ClariCT.AI, ClariPI, Seoul, Republic of Korea) trained by 410,000 image pairs of low- and standard-dose CT from 210 patients and tested with datasets not involved in the training	Retrospective clinical study involving 29 children (mean age: 10.1 y; range: 2–19 y)	19.6% CTDI_vol_ and 14.3% CM concentration reductions in pediatric abdominal DECT with noise-optimized virtual monoenergetic IR when compared with those of standard CT.
Nagayama et al. (2022), Japan [6]	Radiology	DL-Convolutional neural network	DLIR of contrast-enhanced pediatric abdominal CT	CT	Commercially available model (AiCE Body Sharp, Canon Medical Systems) trained by image pairs of lower-dose CT with HIR and high-dose CT with MBIR and tested with datasets not involved in the training	Phantom and retrospective clinical study involving 20 cm diameter Catphan 700 phantom (The Phantom Laboratory, Greenwich, NY, USA) and 65 children (mean age: 25.0 ± 25.2 months; range: 0–81 months), respectively	In pediatric contrast-enhanced 80 kV abdominal CT, 53.7% SSDE reduction with better image quality (e.g., lower noise, noise texture, and edge sharpness improvements, etc.) when compared with standard-dose HIR.
Park et al. (2022), Republic of Korea [7]	Radiology	DL-Generative adversarial network	Post-processing of contrast-enhanced pediatric abdominal CT	CT	Homegrown model trained by 840 unpaired low- (42 patients; mean age: 7.2 ± 2.5 y) and standard-dose (42 patients; mean age: 6.2 ± 2.2 y) pediatric abdominal CT images and validated with 41 datasets (820 images; patient mean age: 7.4 ± 2.2 y) not involved in the training	Retrospective clinical study involving 660 images from 33 children	When compared with standard-dose CT, 36.6% CTDI_vol_ reduction with image noise (7.1 ± 2.7) and CNR (portal vein: 21.2 ± 10.1; liver: 8.5± 4.3) similar to those of SAFIRE images (noise: 9.5 ± 4.0; CNR: 21.2 ± 9.8 (portal vein) and 8.5 ± 5.0 (liver)), and visual assessment (standard-dose and DL-processed image differentiation) yielded a sensitivity and specificity of 61.2% and 35.0%, indicating similar image quality.
Sun et al. (2021), People’s Republic of China and USA [8]	Radiology	DL-Convolutional neural network	DLIR of pediatric neck, chest, and abdominal CT angiography	CT	Commercially available model (TrueFidelity, General Electric Healthcare, Chicago, IL, USA) trained by image pairs of low-dose CT projection (raw) data and higher-dose CT reconstructed by FBP from phantoms and patients	Retrospective clinical study involving 32 children with Takayasu’s arteritis (mean age: 9.1 ± 4.5 y; range: 1–17 y)	High-strength DLIR had highest small artery detection and diagnostic confidence scores based on a 5-point scale (3.53 ± 0.51 and 4.09 ± 0.30) when compared with FBP (2.94 ± 0.25 and 2.91 ± 0.30), ASIR-V 50% (3.03 ± 0.18 and 3.03 ± 0.18), and ASIR-V 100% (2.84 ± 0.37 and 3.00 ± 0.00) groups, respectively, demonstrating its dose reduction potential.
Sun et al. (2021), People’s Republic of China and USA [9]	Radiology	DL-Convolutional neural network	DLIR of pediatric chest CT angiography	CT	Commercially available model (TrueFidelity, General Electric Healthcare) trained by image pairs of low-dose CT projection (raw) data and higher-dose CT reconstructed by FBP from phantoms and patients	Retrospective clinical study involving 33 children (mean age: 5.9 ± 4.2 y; range: 4 months–13 y)	High-strength DLIR images had highest scores of subjective image assessment with a scale of 1–5 (noise: 4.05 ± 0.21 (little); vascular edge: 4.05 ± 0.58 (clear identification); vascular contrast: 4.14 ± 0.64 (good)) when compared with ASiR-V 100% (3.36 ± 0.58; 2.86 ± 0.56; 4.00 ± 0.62) and ASiR-V 50% (2.27 ± 0.55; 3.77 ± 0.61; 3.14 ± 0.64), respectively, demonstrating its potential for further dose reduction.
Sun et al. (2021), People’s Republic of China and USA [10]	Radiology	DL-Convolutional neural network	DLIR of pediatric chest CT angiography	CT	Commercially available model (TrueFidelity, General Electric Healthcare) trained by image pairs of low-dose CT projection (raw) data and higher-dose CT reconstructed by FBP from phantoms and patients	Prospective case-control study involving 54 children (control group: *n* = 27; mean age: 9.5 ± 2.4 y; range: 5–13 y; and study group: *n* = 27; mean age: 9.3 ± 3.1 y; range: 5–14 y)	High-strength DLIR with 70 kV, NI of 22, and CM injection time of 4 s allowed 36% radiation dose and 53% CM dose reductions with scores of subjective image assessment against a 5-point scale similar to control group, ASiR-V 50% with 80 kV, NI of 19, and CM injection time of 8 s (artery contrast: 4.56 vs. 4.78; image quality: 3.67 vs 3.44; diagnostic confidence: 4.74 vs. 4.74; *p* > 0.05).
Sun et al. (2021), People’s Republic of China and USA [11]	Radiology	DL-Convolutional neural network	DLIR of pediatric chest CT angiography	CT	Commercially available model (TrueFidelity, General Electric Healthcare) trained by image pairs of low-dose CT projection (raw) data and higher-dose CT reconstructed by FBP from phantoms and patients	Prospective case-control study involving 92 children (control group: *n* = 46; mean age: 5.9 ± 4.2 y; range: 4 months–13 y; and study group: *n* = 46; mean age: 5.9 ± 4.2 y; range: 4 months–13 y)	High-strength DLIR with 70 kV allowed 11% radiation dose and 20% CM dose reductions with higher scores of subjective image assessment against a 5-point scale (noise: 4 (little); vascular contrast: 4 (good); vascular edge: 4 (clear identification)) when compared with control group, ASiR-V 50% with 100 kV (noise: 2 (high); vascular contrast: 3 (fair); vascular edge: 3 (identifiable)).
Sun et al. (2021), People’s Republic of China and USA [12]	Radiology	DL-Convolutional neural network	DLIR of non-contrast pediatric head CT	CT	Commercially available model (TrueFidelity, General Electric Healthcare) trained by image pairs of low-dose CT projection (raw) data and higher-dose CT reconstructed by FBP from phantoms and patients	Retrospective clinical study involving 50 children (median age: 2 y; range: 0.1–14 y)	High-strength DLIR images with 0.625 mm slice thickness had similar subjective image quality score and measured noise when compared with ASiR-V 50% 5 mm slice thickness images (*p* > 0.05) but able to reduce radiation dose by 85% and improve lesion detection (69 vs. 65 detected) due to spatial resolution increase.
Theruvath et al. (2021), USA [13]	Nuclear medicine	DL-2.5 dimensional encoder-decoder U-Net convolutional neural network	Post-processing of pediatric and adult whole-body PET images	PET/MRI	Commercially available model (SubtlePET 1.3, Subtle Medical, Menlo Park, CA, USA) trained by low- and high-count PET image pairs from whole-body PET/CT and PET/MRI studies of pediatric and adult patients and tested with adult brain and whole-body studies	Prospective clinical study involving 20 pediatric and adult lymphoma patients (mean age: 16.0 ± 6.0 y; range: 6–30 y)	Up to 50% ^18^F-FDG dose reduction with 100% sensitivity and specificity for correct assessment of pediatric and adult lymphoma patients’ treatment response.
Wang et al. (2021), USA and Germany [14]	Nuclear medicine	DL-Convolutional neural network	Post-processing of pediatric and adult ultra-low-dose whole-body PET/MRI images to synthesize standard-dose PET images	PET/MRI	Homegrown model development based on Lim et al.’s [50] open-source enhanced deep super-resolution network model through transfer learning with 17 standard-dose PET, simulated 6.25% ultra-low-dose PET and MRI training datasets, and 6 independent testing datasets acquired in USA	Prospective clinical study involving 34 pediatric and adult lymphoma patients in USA (*n* = 23; mean age: 17 ± 7 y, range: 6–30 y) and Germany (*n* = 11; mean age: 14 ± 5 y; range: 3–18 y)	Expert readers’ agreements of tumor diagnosis between standard and AI-processed 6.25% ultra-low-dose PET images (kappa = 0.942 (USA datasets) and 0.912 (Germany datasets)) were significantly greater than the agreements between standard and 6.25% ultra-low-dose PET images (kappa = 0.650 (USA datasets) and 0.834 (Germany datasets)). Diagnostic accuracy of AI-processed 6.25% ultra-low-dose PET images was adequate, representing 93.75% dose reduction capability.
Yoon et al. (2021), Republic of Korea [15]	Radiology	DL-Convolutional neural network	DLIR of pediatric contrast enhanced abdominal and non-contrast and contrast enhanced chest CT	CT	Commercially available model (TrueFidelity, General Electric Healthcare) trained by image pairs of low-dose CT projection (raw) data and higher-dose CT reconstructed by FBP from phantoms and patients	Phantom and retrospective clinical study involving The Phantom Laboratory’s 20 cm diameter Catphan 500 phantom and 51 pediatric patients (mean age: 11.5 ± 4.6 y; range: 1–18 y), respectively	When compared with ASiR-V 50%, medium- and high-strength DLIR images of contrast enhanced abdominal (*n* = 23) and non-contrast (*n* = 16) and contrast enhanced (*n* = 12) chest CT had statistically significantly higher subjective image quality score and lower noise (*p* < 0.001), illustrating its dose reduction potential.
Zhang et al. (2022), People’s Republic of China [16]	Radiology	DL-Convolutional neural network	DLIR of non-contrast pediatric abdominal and chest CT	CT	Commercially available model (TrueFidelity, General Electric Healthcare) trained by image pairs of low-dose CT projection (raw) data and higher-dose CT reconstructed by FBP from phantoms and patients	Phantom and prospective clinical study involving a pediatric (equivalent to 5-year-old patient) whole body phantom (PBU−70, Kyoto Kagaku Co., Ltd., Kyoto, Japan) and 20 children (mean age: 5.4 ± 1.2 y; range: 4–6 y), respectively	When compared with ASiR-V 70%, high-strength DLIR achieved about 70% and 60% dose reductions for pediatric non-contrast abdominal (*n* = 10) and chest (*n* = 10) CT, respectively. However, high-strength DLIR did not statistically significantly improve subjective image assessment score of chest CT.

^18^F-FDG, fluorine−18-fluorodeoxyglucose; AI, artificial intelligence; AiCE, Advanced Intelligent Clear-IQ Engine; ASiR-V, adaptive statistical iterative reconstruction-Veo; CM, contrast medium; CNR, contrast-to-noise ratio; CT, computed tomography; CTDI_vol_, volume computed tomography dose index; DECT, dual-energy computed tomography; DL, deep learning; DLIR, deep learning image reconstruction; FBP, filtered back projection; HIR, hybrid iterative reconstruction; IR, image reconstruction; MBIR, model-based iterative reconstruction; MRI, magnetic resonance imaging; NI, noise index; No., number; PET, positron emission tomography; PMMA, polymethyl methacrylate; SAFIRE, sinogram affirmed iterative reconstruction; SBIR, statistical-based iterative reconstruction; SSDE, size-specific dose estimate; y, year.

## Data Availability

Not applicable.
